# Identification of key candidates associated with chronic hepatitis E viral infection

**DOI:** 10.6026/973206300210066

**Published:** 2025-01-31

**Authors:** Zoya Shafat, Anam Farooqui, Naaila Tamkeen, Nazim Khan, Asimul Islam, Shama Parveen

**Affiliations:** 1 Centre for Interdisciplinary Research in Basic Sciences, Jamia Millia Islamia, New Delhi, India; 2 Department of Biosciences, Jamia Millia Islamia, New Delhi, India

**Keywords:** Hepatitis E virus, chronic hepatitis E viral infection, differentially expressed genes, enrichment analysis, protein-protein interaction network

## Abstract

An in-depth understanding of chronic hepatitis E viral infection is of interest as the underlying molecular mechanisms remain unexplored. An analysis of mRNA expression profile revealed a total of 69, 157 and 411 Differentially Expressed Genes (DEG) for mild, moderate and severe hepatitis E viral infection, respectively. We found 8 up-regulated genes BATF2, OASL, IFI44L, IFIT3, RSAD2, IFIT1, RASGRP3 and IFI27 having association with persistent hepatitis E viral infection. Of these genes, 6 (OASL, IFI27, IFIT1, IFIT3, RSAD2 and IFI44L) were in protein-protein interaction network and at each stage of infection. Thus, this data provides insights into key genes and linked pathways which could be targeted to offer better interventions for chronic hepatitis E viral infection.

## Background:

Hepatitis E viral (HEV) is a quasi-enveloped virus, with a single-stranded, linear, positive-sense RNA genome [1]. hepatitis E viral is an important cause of waterborne acute hepatitis in adults in developing countries [2, 3]. Previously hepatitis E viral had been reported to have caused acute infections associated with the clearance of the virus. However, Kamar *et al.* reported failure of viral clearance in immunosuppressed patients, *i.e.*, hepatitis E viral patients receiving solid-organ transplant (SOT), leading to chronic hepatitis infections [4]. Chronic hepatitis E viral infection is considered a persistent viral infection as the hepatitis E viral RNA in the patients lasts for more than six months. The chronic hepatitis E viral infection infections were initially reported in 2008 [5, 6], later have been reported by numerous European teams [7, 8-9]. These chronic infections started becoming persistent lasting for more than six months. After kidney transplantation, the incidence of hepatitis E viral infection in patients was estimated to be 2.7 cases/100 person-years [10]. Further, it was recognized that half of the recipients receiving kidney transplantation infected with hepatitis E viral develop chronic hepatitis E viral infection [11]. Moreover, the effective antiviral drug against hepatitis E viral infections is still not available. Chronic hepatitis E viral infection has been well documented in solid-organ transplant recipients; therefore, hepatitis E viral induced chronic hepatitis should be studied intensively. The hepatitis E viral infection evolution toward chronic hepatitis E viral infection seems to be dependent on the patient's immunological status. In solid-organ transplant recipients, the development of chronic hepatitis E viral infection has been linked to the type or dose of immunosuppressive drugs received [12] and one-third of chronic hepatitis E viral infection patients' clear hepatitis E viral after reduction of the dose of immunosuppressive drugs [11]. The mechanisms linked with the development of chronic hepatitis E viral infection are poorly understood. In organ transplant recipients, chronic hepatitis E viral infection has is associated with impaired hepatitis E viral specific T-cell responses [13]. Also, an earlier microarray study has shown the enhanced expression of the interferon-stimulated genes (ISGs), in chimpanzee livers infected with hepatitis E viral suggesting activation of the interferon response by hepatitis E viral [14]. Recently, with the continuous development of bioinformatics and molecular biology, microarray technology has been widely used for exploring the molecular mechanisms of various diseases [15, 16- 17]. Since limited information is available about the prevalence and impact of hepatitis E viral infection in kidney transplant patients, a more detailed study is the need of the hour. Therefore, it is of interest to present to provide insights into the influence of chronic hepatitis E viral infection in kidney trans-plantation. Herein, the original human microarray dataset GSE36539 was accessed from the NCBI-Gene Expression Omnibus database (NCBI-GEO). Following the bioinformatics approach as suggested in previous reports [18, 19]. Therefore, it is of interest to report key candidates associated with chronic hepatitis E viral infection using sequence, structure and function data.

## Materials and Methods:

## Methods flowchart:

The workflow of the integrative network-based method used for the present analysis is illustrated in (Figure 1 - see PDF).

## Microarray data retrieval:

The gene expression dataset GSE36539, deposited by Moal *et al.* was retrieved from the Gene Expression Omnibus gene expression omnibus database (https://www.ncbi.nlm.nih.gov/geo/) of NCBI [20]. The dataset was generated based on the GPL6480 (Agilent-014850 Whole Human Genome Microarray 4x44K G4112F) platform. The experiment contained 8 control samples (kidney transplant recipients without HEV) and 8 infected samples (kidney transplant recipients with HEV) from whole blood tissue. These samples were categorized into three stages of infection, mild (6 samples), moderate (6 samples) and severe (4 samples).

## Data pre-processing:

After GSE36539 was downloaded, probe identification numbers were transformed into gene symbols. For multiple probes corresponding to one gene, the significant expression value was taken as the gene expression value.

## Identification of differentially expressed genes (DEGs):

The GEO2R online tool [21] was used to examine the differential expression of genes. GEO2R is an interactive web tool that compares two groups of samples under the same experimental conditions. The expression prfiles of healthy and infected patients were compared to identify the DEGs. The adjusted P-value (P < 0.05) and a I log_2_ (fold-change) >1 I were set as the inclusion criteria for the genes from each group. To obtain the list of overlapping DEGs, we used Venny 2.1.0, an online tool that can calculate the intersection(s) of listed elements.

## Gene transition among different stages:

To understand the behavior of normal gene expression perturbation, we tried a normal way for finding the associated genes while moving from one stage to another. We identified a list of upregulated and downregulated genes of each transitional stage of infection (*i.e.*, mild, moderate and severe). Studying the genes with differential expression at each stage of infection allows us to get an insight into the on-going mechanobiology inside the cell. In this study, we made a comparison of the gene expression profiles among healthy, mild, moderate and severe stages of infection. These transitions are discussed in brief as follows.

[1] Prodromal phase (mild infection): This segment considers Differentially Expressed Genes which are differentially expressed in between the mildly infected patient group and healthy controls. The prodromal phase includes early disease symptoms like fever, joint pain or arthritis, rash and edema.

[2] Preicteric phase (moderate infection): This section considers Differentially Expressed Genes which are differentially expressed in the moderately infected patient group and healthy controls. The preicteric phase includes the symptoms like myalgia, anorexia, fever, nausea, dark urine, diarrhea, *etc.*.

[3] Icteric phase (severe infection): This section considers Differentially Expressed Genes which are differentially expressed in the severely infected patient group and healthy controls. The icteric phase includes the symptoms like jaundice, anorexia, skin lesions and may subside other symptoms.

## Protein-Protein Interaction (PPI) network analysis:

For further evaluation of the functional interactions among differentially expressed genes, the protein-protein interaction network was constructed from these Differentially Expressed Genes using STRING (Search Tool for the Retrieval of Interacting Genes) [22], which is an online database of known and predicted protein-protein interactions. These interactions include physical and functional associations and the data are mainly derived from computational predictions, high-throughput experiments, automated text mining and co-expression networks. The Differentially Expressed Genes were mapped onto the PPI network and an interaction score of >0.7 was set as the threshold value. Thus, only the interaction pairs with a protein-protein interaction combined score of >0.7 were considered significant. Subsequently, Cytoscape 3.4 [23] was used to visualize and construct the protein-protein interaction network. Nodes with the greatest numbers of interactions with neighboring nodes were considered hub nodes (high degree nodes).

## Functional and pathway enrichment analysis of DEGs:

We used DAVID (The Database for Annotation, Visualization and Integrated Discovery) online server [24] to perform the functional enrichment analysis of differentially expressed genes; this analysis included the functional categories, Gene Ontology (GO) terms and Kyoto Encyclopedia of Genes and Genomes (KEGG) pathways [25, 26]. The GO analysis included 3 categories, namely, biological process (BP), cellular component (CC) and molecular function (MF), which were used to predict protein functions (15). Kyoto Encyclopedia of Genes and Genomes pathway analysis was used to assign sets of Differentially Expressed Genes to specific pathways to enable the construction of the molecular interaction, reaction and relationship networks. Benjamini-adjusted P < 0.05 and an enriched gene count >5 was chosen as the criteria for significance.

## Results:

## Identification of DEGs:

The microarray expression dataset GSE36539 was downloaded from the gene expression omnibus database. The Differentially Expressed Genes between controls and the disease samples were analyzed using the GEO2R tool. The total number of upregulated and downregulated genes were identified between hepatitis E viral infected patients and healthy individuals, using the adjusted P values (P<0.05) and I log_2_FC I >1. The hepatitis E viral patients were distributed into three separate datasets based on the severity of infection: mild, moderate and severe. For each of the datasets, the Differentially Expressed Genes including the upregulated and downregulated genes were identified (Table 1). Comparison of the mild group with the control samples identified 34 upregulated and 35 downregulated genes. Similarly, a comparison of the moderate group with the healthy control samples identified 138 upregulated and 19 downregulated genes. Also, a comparison of the severe group with control identified 326 upregulated and 85 downregulated genes. Thus, a total of 69, 157 and 411 specific Differentially Expressed Genes were identified for mild, moderate and severe hepatitis E viral infections, respectively.

## The interrelationship between stages of infection:

The chronic hepatitis E viral infection patients were sorted into 3 groups: the first category involved patients who experienced early hepatitis E viral clearance (mild infection), *i.e.*, within 6 months after inclusion (median time, 4 months; range, 1.3-4.6 months), the second category involved patients who experienced delayed hepatitis E viral clearance (moderate infection) *i.e.*, >6 months after inclusion (median time, 11.5 months; range, 8.9-17.4 months) and the final category involved patients who did not experience hepatitis E viral clearance (severe infection) during data analysis time (>17.4 months after inclusion). It was noteworthy in Table 1 that with the increase in the severity of the infection, more number of genes got differentially expressed. From Table 1, we tried to find the common genes between these transitional stages. It was found that 9 upregulated genes were commonly differentially expressed among various stages of chronic hepatitis E viral infection (including mild vs. control, moderate vs. control and severe vs. control) (Table 2). However, none of the genes was common in the downregulated differentially expressed genes (Figure 2 - see PDF). The fold change of these genes increases with the increase in the severity of infection. This shows that these 9 genes are the key genes that act from start and with the increase in the upregulation of their gene expression, the infection increases and worsens.

The expression of genes BATF2, OASL, IFI44L, IFIT3, RSAD2, IFIT1, RASGRP3 and IFI27 was significantly higher in patients who did not clear the hepatitis E viral infection as compared to early and later phases of hepatitis E viral infection. However, the expression pattern for the gene OCLN followed a different pattern with the highest expression levels in patients who cleared hepatitis E viral infection at a later stage. The gene expression level in patients with no hepatitis E viral clearance was only slightly higher than patients who cleared the hepatitis E viral infection in early phase. The upper panels of (Figure 3 - see PDF) represent volcano plot of the upregulated and downregulated chronic hepatitis E viral infection genes (Figure 3A - see PDF) while the heat map shows the expression pattern of obtained 8 key genes (BATF2, OASL, IFI44L, IFIT3, RSAD2, IFIT1, RASGRP3 and IFI27) associated with chronic hepatitis E viral infection in different stages (mild, moderate and severe) (Figure 3B - see PDF). The lower panels show the chord plot (Figure 3C - see PDF) depicting the relation between key genes and hepatitis E viral stages of infection. While the correlation heatmap (Figure 3D - see PDF) shows the correlation of the 8 key genes (BATF2, OASL, IFI44L, IFIT3, RSAD2, IFIT1, RASGRP3 and IFI27) with their correlation values.

## Gene term enrichment analysis of degs:

Further, the identified Differentially Expressed Genes were systematically characterized to explore their functions and pathways. The Differentially Expressed Genes were classified into 3 functional categories: biological process (BP), cellular component (CC) and molecular function (MF). The Differentially Expressed Genes that showed significant enrichment are listed in the table (Table 3, Table 4, Table 5). In the molecular function group, the genes were mainly linked to binding and catalytic activities. The moderate and severe gene functions included RNA binding, oligoadenylate synthetase activity, helicase activity, transferase activity, receptor activity, GTP binding, nucleotidyltransferase activity. The mild genes were involved in antigen binding, immunoglobulin receptor binding and RNA polymerase II activating transcription factor binding. In the biological process group, the top GO terms which moderate and severe genes were mainly enriched with included defense response to viruses, negative regulation of viral genome replication, interferon signaling pathways and immune response. In addition, the mild genes were also associated with complement activation, classical pathway, receptor-mediated endocytosis, response to estradiol and response to lithium-ion. In the cellular component group, the mild genes were related to the blood micro particle and mitochondrial outer membrane. The moderate and severe genes were associated with clathrin-coated endocytic vesicle membrane, cytosol, cytoplasm, MHC class II protein complex, an integral component of lumenal side of endoplasmic reticulum membrane.

## Kyoto Encyclopedia of genes and genomes pathway analysis:

For the mild genes group, non-availability of the significantly enriched pathways was observed. The significant signal pathways of moderate and severe genes were mainly enriched with influenza A, Herpes simplex infection, measles, Hepatitis C, RIG-I-like receptor signaling pathway, cytosolic DNA-sensing pathway and *Staphylococcus aureus* infection Table 6. Additionally, the moderate genes were enriched with asthma, rheumatoid arthritis, cytokine-cytokine receptor interaction and severe genes with mineral absorption, antigen processing and presentation and graft-versus-host disease.

## PPI network for transitional stages of infection:

The PPI network with a 0.7 confidence level and no interactors in 1st and 2nd shells was constructed for different stages of infection: (A) mild infection (Figure 4A - see PDF), (B) moderate infection (Figure 4B - see PDF) and (C) severe infection (Figure 4C - see PDF). We identified 6 genes found to be common in all three levels of infection whose expression level increases with the increase in the level of infection (OASL, IFI27, IFIT1, IFIT3, RSAD2, IFI44L). Though previously identified, there were a total of 9 genes common among all three stages, here only 6 of these genes made into the PPI network. It is noteworthy that these six genes form motif with each other. This means that they work together in combination. Surprisingly these genes are low in degree; however they form a motif together. Furthermore, the top nodes were extracted using the Origin 8.0. On the basis of degree centrality, we extracted the top nodes with their degree score (at least 40 degree) from the main network (Figure 5 - see PDF). The Figure 5 (see PDF) shows the seed genes in the decreasing order of their degree with UBA52 possessing the highest degree and GBP1 is having the lowest degree.

## Discussion:

Patients with kidney transplantation have progressively been reported with chronic hepatitis E viral infection since 2008 [5, 6], but the underlying molecular mechanisms leading to the development of this disease remain obscure/unexplored. In this study, transcriptional profiles of the kidney transplant recipients with chronic hepatitis E viral infection were compared with the matched kidney transplant recipients without hepatitis E viral infection for the selection of DEGs. The patients with chronic hepatitis E viral infection were separated into 3 groups according to the time of hepatitis E viral clearance (early, late, or no hepatitis E viral clearance at the time of the analysis). Our analysis revealed a total of 69, 157 and 411 specific Differentially Expressed Genes which included 34 upregulated and 35 downregulated genes, 138 upregulated and 19 downregulated genes and 326 upregulated and 85 downregulated genes for mild, moderate and severe in chronic hepatitis E viral infection respectively. The gene term enrichment analysis of Differentially Expressed Genes associated with mild, moderate and severe stages mostly was associated with signaling processes. More specifically, the top ten Differentially Expressed Genes were mainly involved in interferon and signaling pathways which are consistent with the previous observations made in chronic hepatitis E viral infection patients [27]. The hepatitis E viral genome is organized into three open-reading frames (ORFs), *i.e.*, ORF1, ORF2 and ORF3. The ORF1 polyprotein is further subdivided into multiple domains and is majorly attributed to viral replication. The domains include methyltransferase domain (Met), Y-domain (Y), papain-like-cysteine protease domain (PCP), hyper variable region domain (HVR), X-domain (X), helicase domain (Hel) and RNA-dependent RNA polymerase domain (RdRp) [28]. Previous investigations have explored some of the domains that suggested their functional implications, such as, transferase activity, RNA binding, helicase activity, zinc-ion binding and protein binding as major molecular functions. The Met domain has been suggested as a putative methyltransferase [29]. A highly conserved α-helix counterpart 'LYSWLFE' (aa 410-416) has been predicted in the Y-domain, required for its cytoplasmic membrane binding and efficient replication [30]. The presence of potential Appr1"-pase active site (Asn806, Asn809, His812, Gly815, Gly816 and Gly817) in the X-domain has demonstrated its significant catalytic/regulatory function in hepatitis E viral replication [31, 32]. The Pro domain has been shown to be involved in the disorder-to-order state transition required for binding to various substrates. Also, earlier it has been suggested that Pro domain is not required for the replication of a viral and its infectivity, but performs a role in replication efficiency [33, 34]. The Hel domain consists of two conserved motifs: Walker A (GVPGSGKS; aa 975-982) and Walker B (DEAP; aa 1029-1032), which has been demonstrated to participate in purine nucleoside triphosphate (NTP)-binding activity [35, 36]. The RdRp protein found in ORF1 OF hepatitis E viral (positive sense) is necessary for its genome replication [37, 38]. The ORF2 encodes the capsid protein which forms the hepatitis E viral virion major structural component [39]. ORF2 (110 amino acids at N-terminus) interacts with the 5' region (encapsidation signal) of hepatitis E viral genomic RNA [40].

Additionally, ORF2 N-terminus entails a signal peptide followed by an arginine-rich domain, which has shown involvement in viral RNA encapsidation during the assembly process [41]. ORF3 is a phosphoprotein consisting of about 113 to 114 amino acid residues that perform a crucial function in the viral egress or release from infected cells [42, 43]. The ORF3 protein has been demonstrated to participate in interaction with several host proteins in addition to interaction with the ORF2 protein [44]. Additionally, the proline-rich motif (PSAP) in the C-terminal region has been reported to play a role in the activity of ESCRT machinery by interacting with Tsg101 [45]. Thus, these predicted functions further substantiate our findings. Interestingly, in our study, we found the upregulated expression levels of 8 genes BATF2, OASL, IFI44L, IFIT3, RSAD2, IFIT1, RASGRP3 and IFI27. The expression of these genes was significantly higher in chronic hepatitis E viral infection patients who did not clear the hepatitis E viral infection as compared to chronic hepatitis E viral infection patients with early hepatitis E viral clearance. Also, it was revealed that the gene expression level in chronic hepatitis E viral infection patients who had late hepatitis E viral clearance was intermediate between the hepatitis E viral patients with early clearance and no hepatitis E viral clearance. BATF2 is a transcription factor and is involved in T cell receptor signaling pathway [46, 47]. BATF2 regulates the expression of target genes by binding as a heterodimer on recognizing the target DNA sequence. BATF2 (SARI) was found to be upregulated in chronic hepatitis E viral infection patients in an earlier study [27]. OASL is a gene known to encode proteins known to be antiviral effectors and is mainly linked with signaling pathways [48]. Upregulated expression of the OASL has previously been reported in chronic hepatitis E viral infection patients [27]. IFI44L, a paralog of IFI44, has been reported to encode a protein that induces interferons. IFI44L a 44-kD protein originally identified as the HCV-associated microtubular aggregate in the hepatocytes of chimpanzees infected with HCV [49]. IFI44L has also been demonstrated with high anti-HCV antiviral activity [50]. RSAD2 (viperin) has been reported as one of the most highly induced interferon effector proteins [51]. Previous investigation has shown the upregulated expression of RSAD2 in chronic hepatitis E viral infection patients [27]. RASGRP3 is known to encode a guanine nucleotide exchange factor protein. IFIT1 and IFIT3 genes belonging to the IFIT complex are known to antagonize the viruses by their nucleic acid sequestration. Earlier, the upregulated expression of IFIT complex has been documented in chronic hepatitis E viral infection patients [27]. IFI27 codes for interferon-inducing protein. It functions in TNFSF10-induced apoptosis and is also involved in the interferon-induced negative regulation of the transcriptional activity. However, the OCLN gene had a peculiar characteristic gene expression pattern from the other upregulated genes. OCLN gene is an essential entry factor in HCV cell and encodes a membrane protein and is involved in cytokine-induced regulation [52]. Taken together, these findings from the present study propose that the association of these upregulated genes BATF2, OASL, IFI44L, IFIT3, RSAD2, IFIT1, RASGRP3 and IFI27 is associated with persistent hepatitis E viral infection. Also, it was important to discuss that though our study identified a total of 9 genes that were common among three stages, but only 6 of them (OASL, IFI27, IFIT1, IFIT3, RSAD2 and IFI44L) made into the PPI network whose expression level increases with the increase in the level of infection. Thus, these selected 6 genes were found to be common at each stage of infection. It is noteworthy to mention that these six genes formed motif with each other and thus work together in combination. To sum up our results, we can conclude that patients with no hepatitis E viral clearance had significantly higher hepatitis E viral loads than patients who cleared the hepatitis E viral infection, irrespective of early or late stages. This clearly indicates that hepatitis E viral loads are associated with the timing of hepatitis E viral and is in agreement with the previous investigation carried out in chronic hepatitis E viral infection patients [27]. Previous microarray-based report offered significant insight into the pathogenesis of hepatitis E viral associated with persistent hepatitis E viral infection. It is worth mentioning that some of the upregulated genes discovered in our analysis presented the same genes as in the previous report that demonstrated the upregulation of EPSTI1, ISG15, IFIT1, IFI44L and RSAD2 [27]. However, our study captured other additional genes BATF2, OASL, IFIT3, RASGRP3 and IFI27 and explored the other important aspect of genes specific to stage transition of hepatitis E viral Further studies are envisaged to explicate the functional role of these upregulated genes in chronic hepatitis e viral infection These findings further support the hypothesis proposed by the current study.

## Conclusions:

Our understanding of chronic hepatitis E viral infection pathophysiology has improved through this analysis. However, exploration of possible mechanisms for chronic hepatitis E viral infection is warranted.

## Declarations:

## Availability of data and material:

Not applicable

## Funding:

Not applicable

## Figures and Tables

**Table 1:** The Differentially Expressed Genes identified associated with CHE

**Mild v/s Control**	
**Upregulated**	**Down regulated**
BATF2, OASL, IFI44L, FAM83A, LINC00900, DUSP26, APBB2, NAPSA, NAPSA, SLC45A3, IFIT3, NAPSB, CXXC5, FCRL5, RSAD2, EBF1, LY6K, IGFBP2, HIC1, OR5T2, IFIT1, USH1G, RASGRP3, GLYATL2, HIC1, OCLN, IGHA1, TTC7B, IGK///IGKV3-20///IGHV3-23, FNIP1, IGSF22, IFI27, IGKV1-5	THEM4, LOC389831, TVP23C-CDRT4, KANSL1-AS1, TIGD7, LPIN1, THEM4, FAM13A, KRTCAP3, HERC2P7, LOC389831, C17orf100, MXRA7, ZNF528, LOC283788, NBPF1, SYT15, PEX6, EPB41L5, CSGALNACT1, PTGS2, SNAPC1, ZNF181, GSTT1, ATP6V0E2-AS1, PCBP1-AS1, AVIL, PAQR8, CCDC77, CD200R1, GOLGA6L6, AK5, TMEM45B, IGIP
**Moderate v/s Control**	
**Upregulated**	**Down regulated**
ISG15, USP18, IFI44L, RSAD2, USP18, RSAD2, HERC5, OAS3, LY6E, IFI6, HERC6, IFIT1, XAF1, IFIT3, MX1, OAS2, OASL, EIF2AK2, DDX58, TRIM22, PARP12, BATF2, IFI44, XAF1, IFIT5, IFIH1, OAS3, SERPING1, IFIT5, SPATS2L, DDX60, DDX60, DHX58, TRIM22, PARP9, OAS2, OCLN, SAMD9L, MT2A, EPSTI1, SIGLEC1, GBP1, GBP1, IRF7, SAMD9, XAF1, TRIM6, HERC6, MT2A, LAMP3, ETV7, DOCK4, RTP4, DDX60L, SLC39A8, SAMD9, OCLN, SIGLEC1, APOBEC3B, DOCK4, TRIM6, EPSTI1, IFI27, AMD9, CXCL11, OAS1, CD38, ZCCHC2, PNPT1, SAMD9, MX2, AIM2, CXCL11, IFIT2, IFI27, ERAP2, OCLN, APOBEC3A, TNFSF10, C3AR1, IRF7, CEP55, GBP3, MS4A4A, NCOA7, IFIT2, CX3CR1, KNOP1, PLSCR1, GBP5, TNFSF15, CDKN1C, HPGD, NOC3L, FCGR1B, PTPRO, HESX1, CRYZ, RHOBTB3, LOC100507460, MANEA, SPIC, PAX8-AS1, CTSL, LOC339803, AGRN, PAX8-AS1, IDO1, TNFRSF11A, ZBP1, MS4A2, PPM1K, CMKLR1, ZNF100, LPAR6, FGF7, RASGRP3, SLC12A8, CXCL10, NCOA7, FAM83A, CCR5, DUSP26, ZBP1, KLHDC7B, HCFC2, ANKRD22, CTAG1A, PTPN13, GPR20, KBTBD3, CMKLR1, USH1G, ERAP2, L1TD1, POM121L1P, NUDT12	RPS26, RPS26, RPS26, NQO2, HLA-DQA2, HLA-DQA1, ZNF667-AS1, LOC644450, QKI, SAPCD2, CD1C, ZNF467, ORM2, TUBB8, HLA-DQB1, TEKT4, ORM1, MXRA7
**Severe v/s Control**	
**Upregulated**	**Down regulated**
USP18, USP18, ISG15, IFI44L, XAF1, OAS3, RSAD2, OAS2, RSAD2, LY6E, HERC5, IFI6, HERC6, EIF2AK2, BATF2, SERPING1, IFIT1, XAF1, IFIT3, OASL, PML, IFI35, DTX3L, OAS2, GBP4, PARP14, MX1, DDX58, IFIH1, PARP9, MOV10, OAS3, GBP1, PARP12, SPATS2L, ABTB2, PARP14, IFIT5, XRN1, GBP1, EPSTI1, TRIM22, TOR1B, HERC6, DDX60, IRF7, DHX58, UBE2L6, TRIM6, AIM2, IFIT5, DDX60, STAT1, FBXO6, IFI44, TAP1, PARP14, GBP5, ETV7, RNF213, TDRD7, SAMD9, XAF1, TRIM6, PARP14, SAMD9L, PML, MT2A, PNPT1, LAMP3, IFI27, TRIM22, GRIN3A, MT2A, NEXN, MT1X, PSMB9, PHF11, MT1X, GBP4, MT1B, IFIT2, EPSTI1, MT1H, MT1G, SAMD9, MT2A, OAS1, SIGLEC1, ZCCHC2, RTP4, APOBEC3B, DNAH3, NEXN, IDO1, SAMD9, ZBP1, ZCCHC2, IFI27, DOCK4, GBP2, ZC3HAV1, IFIT2, MT1H, SIGLEC1, ANKRD22, MX2, DDX60L, CYSLTR1, IRF7, NT5C3A, ZC3HAV1, DOCK4, SP110, ZNFX1, KIAA1958, FCGR1B, TNFSF10, LGALS9, SAMD9, CD274, TRAFD1, AGRN, CD38, RMI2, PTGFR, CMTR1, LOC100419583, GTPBP2, PML, MT1L, SLFN5, TAP2, ZCCHC2, MT1E, MX2, CCL2, FAS, STAT2, APOBEC3A, DTX3L, APOL2, PML, ZBP1, DDX60L, SECTM1, TNFSF13B, AGRN, TRIM14, KIAA0319L, EXOC3L1, C18orf25, Sep-04, DBF4B, ZC3HAV1, IFI16, LOC107987020, IFI16, C19orf66, CXCL11, ANKFY1, PLSCR1, NCF1, DYNLT1, SERPINB9, RERE, HESX1,CPT1B, LYSMD2, FLJ42393, APOL2, DISC1, NAGK, ADAR, RBM43, DISC1, NMI, NCOA7, NCF1, RASGRP3, HOXB3, GPR84, FANCA, LINC01410, FAS, CHMP5, SMCHD1, TIFA, EPHB2, PML, STAT1, ITSN1, TRIM38, ZNFX1, TAF8, KIAA0319L, CCR1, TMEM123, IFITM3, LOC100509780, DDAH1, TRIM5, RBM47, TREX1, LHFPL2, GRAMD1B, XRN1, PPM1K, UBQLNL, SP100, SECTM1, C2, TMEM123, DDX17, TRIM34, POM121L1P, POM121L4P, ZNF496, DISC1, OR52K3P, TRANK1, REC8, ZNF366, HLX, PAPLN, C1GALT1, CCR1, GALM, APOL6, GBP3, FAM225B, IRF9, DYSF, RBP4, DOCK4, SNTB1, ERAP2, STAP1, CXCL11, SP140, AP5B1, DPY19L1P1, TYMP, TRANK1, POM121L4P, TACSTD2, NCOA7, AFAP1-AS1, SPIC, JUP, HSPA6, ATOH8, PPM1K, SHISA5, TTC21A, OR52K2, CXorf21, PNPT1, HSH2D, APOL1, TRIM5, GRB10, FAM8A1, ZNF684, CYP2C19, EDNRB, IFITM4P, LAP3, KPTN, CXCL10, RASL11A, BST2, TRIM21, SAMD4A, KLHDC7B, PAM, CD300E, RNF144A, HPR, MICB, SPATA16, OCLN, TRIM14, PML, C1QB, GBP3, CARD6, NCOA7, C3AR1, FKBPL, SF3B3, TMEM27, ARG2, IL4I1, SIPA1L2, FUT10, SEC24D, DBIL5P2, SLC26A8, GIMAP8, IFITM1, IGF2BP3, AFF1, PGAP1, RBM43, PTK2, IFITM2, NNAT, PARP11, PHC2, CDC42EP2, TNNT2, C5orf56, SMAD1, PAX8-AS1, LGALS3BP, KIAA0319L, CD274, RAB12, C1QC, APOL6, PGAP1, GIMAP8, DNAH17, PARP11	FAM101B, EIF4EBP2, GPX7, UNC119B, ANKS6, GPX7, HKR1, RPL23AP7, RPL23AP87, ZNF395, MEI1, HLA-DQA1, KIAA1147, NBPF3, RPL23AP87, DLG5, EIF1AY, PABPC3, AGAP1, PITPNM2, DDHD2, MXRA5, TSTD3, TMEM97, RPL23AP32, ZNF467, KIF5A, HLA-DQB1, ZSWIM5, CLIC5, FGL1, GSTT1, SLC26A11, EIF4B, RPL23AP7,CRACR2B, EIF4EBP2, ID3, SET, DRG2, RPS11P6, RPS10P7, RNF113B, PGM5-AS1, ISCA1, FBXL16, TPT1P8, NAP1L1, C16orf74, RPL22, TMEM30B, EEF1DP4, ZFYVE28, KLF12, ANKS6, HLA-DQA2, LOC155060, NACAP1, HERC2P7, PFN1P2, RPL24, KDM5D, GOLGA6C, LOC100131170, CSGALNACT1, HLA-DQB2, GJC3, ME3, FADS2, LINC00265, WDR76, TXLNGY, DGKH, HNRNPA1, FAM133B, GOLGA2P7, OLFM1, ANKH, LINC00675, GSTM4, HNRNPA1L2, RPS4Y2, GDA, ARVCF

**Table 2:** Common genes in mild, moderate and severe infection

**Gene Symbol**	**Gene Name**	**Log FC in mild infection**	**Log FC in moderate infection**	**Log FC in severe infection**
BATF2	Basic leucine zipper ATF-like transcription factor 2	1.2	2.043893	3.655921
IFI44L	Interferon induced protein 44 like	1.32	4.017561	5.286781
IFIT3	Interferon induced protein with tetratricopeptide repeats 3	1.01	2.81653	4.020288
RSAD2	Radical S-adenosyl methionine domain containing 2	1.01	3.878622	5.45379
IFIT1	Interferon induced protein with tetratricopeptide repeats 1	1.26	3.851601	4.993007
RASGRP3	RAS guanyl releasing protein 3	1.13	1.156208	2.031469
OCLN	Occludin	1.05	2.534	1.455377
IFI27	Interferon alpha inducible protein 27	1.86	4.086951	5.38789

**Table 3:** Gene term enrichment analysis of Differentially Expressed Genes associated with mild conditions

**Term**	**Description**	**Genes**	**Count**	**P-value**	**FPC**
		**Biological Process**			
GO:0060337	Type I interferon signaling pathway	IFI27, RSAD2, IFIT1, IFIT3, OASL	5	2.12E-05	0.005591
GO:0051607	Defense response to virus	RSAD2, IFIT1, IFI44L, IFIT3, OASL	5	8.23E-04	0.10863
GO:0006958	Complement activation, classical pathway	IGHV3-23, IGKV1-5, IGHA1, IGKV3-20	4	0.002068	0.181942
GO:0009615	Response to virus	RSAD2, IFIT1, IFIT3, OASL	4	0.00279	0.18413
GO:0045071	Negative regulation of viral genome replication	RSAD2, IFIT1, OASL	3	0.004697	0.248004
GO:0050776	Regulation of immune response	CD200R1, IGHV3-23, IGKV1-5, IGKV3-20	4	0.010592	0.449752
GO:0006898	Receptor-mediated endocytosis	IGHV3-23, IGKV1-5, IGHA1, IGKV3-20	4	0.011925	0.449752
GO:0006956	Complement activation	IGHV3-23, IGKV1-5, IGKV3-20	3	0.020883	0.60238
GO:0032355	Response to estradiol	IFI27, IGFBP2, PTGS2	3	0.022713	0.60238
GO:0010226	Response to lithium ion	IGFBP2, PTGS2	2	0.022817	0.60238
		**Molecular Function**			
GO:0003823	Antigen binding	IGHV3-23, IGKV1-5, IGHA1, IGKV3-20	4	0.00228	0.223479
GO:0034987	Immunoglobulin receptor binding	IGHV3-23, IGHA1	2	0.064208	1
GO:0001102	RNA polymerase II activating transcription factor binding	IFI27, DUSP26	2	0.092467	1
		**Cellular Component**			
GO:0072562	Blood microparticle	IGHV3-23, IGKV1-5, IGHA1, IGKV3-20	4	0.009327	0.727468
GO:0005741	Mitochondrial outer membrane	IFI27, RSAD2, LPIN1	3	0.067529	1

**Table 4:** Gene term enrichment analysis of Differentially Expressed Genes associated with moderate conditions

**Term**	**Description**	**Genes**	**Count**	**P-value**	**FDC**
		**BIOLOGICAL PROCESS**			
GO:0051607	Defense response to virus	RSAD2, MX2, MX1, IFIT5, EIF2AK2, ISG15, IFIT1, DDX60, IFIT3, IFI44L, IFIT2, OASL, HERC5, CXCL10, PLSCR1, OAS1, OAS2, OAS3, DHX58, GBP1, TRIM22, APOBEC3A, GBP3, APOBEC3B	24	3.68E-25	1.93E-22
GO:0009615	Response to virus	RSAD2, DDX58, MX2, MX1, IFI44, EIF2AK2, IFIT1, DDX60, IFIT3, IFIT2, OASL, IFIH1, OAS1, OAS2, OAS3, DHX58, IRF7, TRIM22	18	1.47E-19	3.85E-17
GO:0060337	Type I interferon signaling pathway	RSAD2, MX2, MX1, IFI6, ISG15, IFIT1, IFIT3, IFIT2, OASL, IFI27, OAS1, OAS2, OAS3, IRF7, XAF1	15	1.56E-18	2.73E-16
GO:0045071	Negative regulation of viral genome replication	TRIM6, PLSCR1, RSAD2, OAS1, OAS3, MX1, EIF2AK2, ISG15, IFIT1, APOBEC3A, OASL	11	3.51E-14	4.61E-12
GO:0060333	Interferon-gamma-mediated signaling pathway	MT2A, OAS1, OAS2, OAS3, IRF7, FCGR1B, GBP1, HLA-DQA2, TRIM22, HLA-DQA1, OASL, HLA-DQB1	12	5.05E-13	5.30E-11
GO:0006955	Immune response	TNFSF15, IFI6, CXCL10, CXCL11, AIM2, OAS1, OAS2, OAS3, TNFSF10, CCR5, MS4A2, FCGR1B, HLA-DQA2, TRIM22, HLA-DQA1, HLA-DQB1, CMKLR1	17	5.61E-09	4.91E-07
GO:0045087	Innate immune response	ZBP1, DDX58, MX2, MX1, IFIT5, EIF2AK2, DDX60, IFIH1, HERC5, AIM2, DHX58, IRF7, SERPING1, APOBEC3A, APOBEC3B	15	3.64E-07	2.73E-05
GO:0032480	Negative regulation of type I interferon production	IFIH1, HERC5, DDX58, DHX58, ISG15	5	3.24E-05	0.002124
GO:0006954	Inflammatory response	GBP5, CXCL10, CXCL11, ORM1, AIM2, C3AR1, SIGLEC1, TNFRSF11A, CCR5, MS4A2	10	5.36E-04	0.031277
GO:0006935	Chemotaxis	CX3CR1, CXCL10, CXCL11, C3AR1, CCR5, CMKLR1	6	9.25E-04	0.045172
		**MOLECULAR FUNCTION**			
GO:0003725	Double-stranded RNA binding	IFIH1, OAS1, DDX58, OAS2, OAS3, DHX58, EIF2AK2, DDX60, OASL	9	4.13E-09	8.14E-07
GO:0001730	2'-5'-oligoadenylate synthetase activity	OAS1, OAS2, OAS3, OASL	4	9.58E-07	9.44E-05
GO:0003727	Single-stranded RNA binding	IFIH1, DDX58, DHX58, IFIT5, L1TD1, DDX60	6	7.07E-06	4.64E-04
GO:0004386	Helicase activity	IFIH1, DDX58, DHX58, DDX60L, DDX60	5	0.002013	0.099154
GO:0016740	Transferase activity	OAS1, OAS2, OAS3, CD38, OASL	5	0.003134	0.123478
GO:0032395	MHC class II receptor activity	HLA-DQA2, HLA-DQA1, HLA-DQB1	3	0.003888	0.12766
GO:0004950	Chemokine receptor activity	CX3CR1, CCR5, CMKLR1	3	0.004995	0.140574
GO:0005525	GTP binding	TUBB8, GBP5, MX2, MX1, RHOBTB3, GBP1, IFI44L, GBP3	8	0.010617	0.261449
GO:0016779	Nucleotidyltransferase activity	OAS1, OAS2, OAS3	3	0.012376	0.270907
GO:0003924	GTPase activity	TUBB8, GBP5, MX2, MX1, GBP1, GBP3	6	0.016042	0.316029
		**CELLULAR COMPONENT**			
GO:0030669	Clathrin-coated endocytic vesicle membrane	FCGR1B, HLA-DQA2, HLA-DQA1, HLA-DQB1	4	0.002084	0.168858
GO:0005829	Cytosol	DOCK4, HPGD, RHOBTB3, IFIT1, USP18, IFIT3, CRYZ, IFIT2, OASL, IFIH1, HERC5, MT2A, TRIM6, GBP1, TRIM22, HERC6, ZBP1, DDX58, MX2, MX1, EIF2AK2, ISG15, PARP9, RPS26, PLSCR1, AIM2, OAS1, USH1G, OAS2, OAS3, IRF7, XAF1, IDO1	33	0.005355	0.168858
GO:0005887	Integral component of plasma membrane	CX3CR1, TNFSF15, PTPRO, GPR20, TNFRSF11A, CD1C, RASGRP3, PLSCR1, LPAR6, TNFSF10, C3AR1, SLC39A8, CCR5, MS4A2, HLA-DQA2, HLA-DQA1, CMKLR1, LY6E	18	0.00626	0.168858
GO:0005737	Cytoplasm	CDKN1C, RTP4, SAMD9, HPGD, IFIT1, DDX60, IFI44L, IFIT3, CRYZ, IFIT2, OASL, TUBB8, HERC5, MT2A, TRIM6, CTAG1A, DHX58, TEKT4, CCR5, TRIM22, HERC6, FAM83A, ZBP1, NQO2, PNPT1, DDX58, MX2, MX1, IFI44, EIF2AK2, DUSP26, PARP9, PTPN13, HCFC2, QKI, RPS26, AIM2, OAS1, SAPCD2, OAS2, OAS3, IRF7, SPATS2L, AGRN, ZCCHC2, APOBEC3A	46	0.006836	0.168858
GO:0042613	MHC class II protein complex	HLA-DQA2, HLA-DQA1, HLA-DQB1	3	0.008118	0.168858
GO:0071556	Integral component of lumenal side of endoplasmic reticulum membrane	HLA-DQA2, HLA-DQA1, HLA-DQB1	3	0.013872	0.221275
GO:0032588	Trans-Golgi network membrane	RHOBTB3, HLA-DQA2, HLA-DQA1, HLA-DQB1	4	0.014894	0.221275
GO:0030658	Transport vesicle membrane	HLA-DQA2, HLA-DQA1, HLA-DQB1	3	0.023168	0.301189
GO:0010008	Endosome membrane	IRF7, CD1C, HLA-DQA2, HLA-DQA1, HLA-DQB1	5	0.028155	0.318493
GO:0032421	Stereocilium bundle	DOCK4, IDO1	2	0.030624	0.318493

**Table 5:** Gene term enrichment analysis of Differentially Expressed Genes associated with severe conditions

**Term**	**Description**	**Genes**	**Count**	**P-value**	**FDR**
		**BIOLOGICAL PROCESS**			
GO:0051607	Defense response to virus	IFITM3, IFITM1, IFITM2, IFIT5, ADAR, ZC3HAV1, IFIT1, DDX60, IFI44L, IFIT3, IFIT2, OASL, HERC5, IFI16, TRIM5, DHX58, GBP1, TRIM22, GBP3, RSAD2, STAT1, STAT2, MX2, MX1, EIF2AK2, ISG15, PML, BST2, CXCL10, PLSCR1, OAS1, OAS2, OAS3, C19ORF66, IRF9, APOBEC3A, TRIM34, APOBEC3B	38	6.52E-32	8.20E-29
GO:0060337	Type I interferon signaling pathway	IFITM3, IFITM1, SP100, IFITM2, IFI6, ADAR, IFI35, IFIT1, IFIT3, IFIT2, OASL, GBP2, RSAD2, STAT1, STAT2, MX2, MX1, ISG15, BST2, OAS1, IFI27, OAS2, OAS3, IRF7, XAF1, IRF9	26	1.15E-28	7.24E-26
GO:0060333	interferon-gamma-mediated signaling pathway	SP100, STAT1, NMI, PML, OASL, MT2A, OAS1, OAS2, TRIM5, OAS3, IRF7, TRIM38, GBP2, FCGR1B, GBP1, HLA-DQA2, TRIM21, TRIM22, IRF9, HLA-DQA1, HLA-DQB2, HLA-DQB1, TRIM34	23	9.43E-23	3.96E-20
GO:0045071	negative regulation of viral genome replication	IFITM3, IFITM1, IFITM2, RSAD2, MX1, EIF2AK2, ISG15, ADAR, ZC3HAV1, IFIT1, OASL, BST2, PLSCR1, TRIM6, IFI16, OAS1, OAS3, C19ORF66, APOBEC3A	19	2.54E-22	7.98E-20
GO:0009615	response to virus	IFITM3, IFITM1, IFITM2, RSAD2, DDX58, MX2, MX1, IFI44, EIF2AK2, ADAR, ZC3HAV1, IFIT1, DDX60, IFIT3, IFIT2, OASL, IFIH1, BST2, OAS1, OAS2, OAS3, DHX58, IRF7, TRIM22	24	1.86E-19	4.69E-17
GO:0035455	response to interferon-alpha	IFITM3, BST2, IFITM1, IFITM2, LAMP3, MX2, EIF2AK2, ADAR	8	3.04E-11	6.37E-09
GO:0045087	innate immune response	C1QB, NCF1, IFIT5, ADAR, ZC3HAV1, DDX60, C2, IFIH1, HERC5, IFI16, TRIM5, DHX58, APOL1, TRIM21, ZBP1, DDX58, MX2, MX1, EIF2AK2, PTK2, PML, BST2, AIM2, IRF7, SERPING1, TRIM14, CD300E, APOBEC3A, C1QC, APOBEC3B	30	7.96E-11	1.43E-08
GO:0035456	response to interferon-beta	IFITM3, BST2, IFITM1, PLSCR1, IFITM2, STAT1, XAF1	7	1.35E-09	2.12E-07
GO:0045926	negative regulation of growth	MT2A, PNPT1, MT1L, MT1G, MT1H, MT1X, MT1B, MT1E	8	1.13E-08	1.58E-06
GO:0006955	immune response	IFITM3, CD274, IFITM2, IFI6, SECTM1, TNFSF13B, TNFSF10, CCL2, GBP2, FCGR1B, HLA-DQA2, TRIM22, HLA-DQA1, CCR1, SERPINB9, CXCL10, CXCL11, AIM2, OAS1, OAS2, OAS3, FAS, HLA-DQB2, C1QC, HLA-DQB1	25	9.33E-08	1.17E-05
		**MOLECULAR FUNCTION (MF)**			
GO:0008270	Zinc ion binding	RERE, SP100, KDM5D, GDA, MT1L, SP140, PAPLN, MT1X, DBF4B, IFIH1, MT2A, TRIM6, RNF213, RNF113B, TRIM5, DHX58, TRIM21, ZSWIM5, TRIM22, PHC2, DTX3L, ERAP2, DDX58, SP110, PHF11, PML, RNF144A, ZNFX1, OAS1, OAS2, MT1G, TRIM14, MT1H, TRIM38, MT1B, SEC24D, XAF1, PAM, ZCCHC2, APOBEC3A, MT1E, TRIM34, APOBEC3B	43	7.06E-07	2.71E-04
GO:0003725	Double-stranded RNA binding	IFIH1, OAS1, DDX58, OAS2, OAS3, DHX58, EIF2AK2, DDX60, OASL	9	5.61E-06	0.001077
GO:0001730	2'-5'-oligoadenylate synthetase activity	OAS1, OAS2, OAS3, OASL	4	1.62E-05	0.00207
GO:0004386	Helicase activity	IFIH1, DDX17, MOV10, DDX58, DHX58, DDX60L, DDX60, EIF4B	8	4.32E-04	0.032951
GO:0004871	Signal transducer activity	BST2, JUP, STAT1, SP110, STAT2, SHISA5, FAS, SECTM1, TRIM38, LGALS9, PTK2, RASGRP3	12	4.73E-04	0.032951
GO:0003723	RNA binding	ZBP1, DDX17, PNPT1, SF3B3, RPL22, IFIT5, SAMD4A, ADAR, DDX60L, IFIT1, IFIT3, IFIT2, RPL24, PABPC3, IGF2BP3, C19ORF66, HNRNPA1, TRIM21, HNRNPA1L2, RBM43, EIF4B	21	5.62E-04	0.032951
GO:0003727	Single-stranded RNA binding	IFIH1, DDX58, DHX58, IFIT5, DDX60, HNRNPA1	6	6.01E-04	0.032951
GO:0016740	Transferase activity	OAS1, OAS2, OAS3, CD38, GSTT1, OASL	6	0.019243	0.615791
GO:0016874	Ligase activity	RNF144A, HERC5, TRIM6, C18ORF25, RNF213, DTX3L, TRIM5, TRIM38, TRIM21, TRIM22	10	0.030843	0.909973
GO:0003924	GTPase activity	GBP5, MX2, MX1, GTPBP2, SEPT4, GBP2, GBP1, GBP4, GBP3	9	0.035736	0.909973
		**CELLULAR COMPONENT**			
GO:0005829	Cytosol	DOCK4, NCF1, GDA, ITSN1, UBE2L6, IFI35, IFIT1, GIMAP8, IFIT3, OASL, IFIT2, ZFYVE28, IFIH1, HERC5, MT2A, TRIM6, TRIM5, KIF5A, GRB10, ANKFY1, FBXO6, EPHB2, TRIM21, TRIM22, HERC6, ZBP1, FKBPL, DTX3L, DDX58, RPL22, SERPINB9, PARP9, NT5C3A, RBP4, HSH2D, PLSCR1, AIM2, DDAH1, XRN1, OAS1, OAS2, OAS3, TNNT2, CDC42EP2, IRF7, RPL24, IRF9, IDO1, CHMP5, UNC119B, SET, TACSTD2, GSTT1, NDRG2, USP18, TYMP, RNF213, IFI16, IGF2BP3, GBP2, GBP1, EIF4B, GSTM4, SMAD1, JUP, STAT1, TREX1, STAT2, MX2, HSPA6, MX1, DDHD2, EIF2AK2, ISG15, PTK2, PML, PSMB9, MOV10, NAGK, FAS, TRIM38, SEC24D, XAF1, TRIM34	84	2.06E-06	4.82E-04
GO:0048471	Peri-nuclear region of cytoplasm	SET, MT1L, MT1X, NDRG2, TNFSF13B, RASGRP3, HERC5, MT2A, LAMP3, KIF5A, CCL2, GBP2, DISC1, GBP4, GBP3, STAT1, MX1, EIF2AK2, OAS2, TAF8, MT1G, MT1H, MT1B, SEC24D, KPTN, PAM, MT1E	27	4.21E-06	4.92E-04
GO:0005737	Cytoplasm	NCF1, MT1L, MT1X, IFIT1, IFIT3, GIMAP8, IFI44L, IFIT2, HERC5, MT2A, DHX58, GRB10, FBXO6, LGALS9, CMTR1, TRIM21, TRIM22, HERC6, ZBP1, RPL22, XRN1, RPL24, ANKS6, TRIM14, MT1G, MT1H, C19ORF66, LAP3, MT1B, DGKH, MT1E, PTGFR, ZNF395, SET, AGAP1, ZC3HAV1, DDX60, NDRG2, TMEM27, RNF213, CRACR2B, STAP1, EIF4EBP2, GSTM4, SMAD1, PNPT1, JUP, DNAH17, TPT1P8, HSPA6, FANCA, IFI44, EIF2AK2, TDRD7, NMI, PARP14, PTK2, BST2, ABTB2, DLG5, ID3, SPATS2L, HNRNPA1L2, ZCCHC2, APOBEC3A, RTP4, CLIC5, ADAR, TNFSF13B, OASL, TRIM6, KIAA1147, TRIM5, KLF12, RMI2, NBPF3, DTX3L, DDX58, SERPINB9, PARP9, NT5C3A, AIM2, OAS1, OAS2, OAS3, IRF7, CDC42EP2, PABPC3, IRF9, DNAH3, SP100, SAMD9, DBF4B, ARVCF, APOL6, IFI16, ATOH8, NNAT, IGF2BP3, HNRNPA1, STAT1, STAT2, MX2, MX1, DDHD2, FBXL16, PML, PSMB9, FAS, GALM, KIAA1958, AGRN, SNTB1, TRIM34	114	1.84E-05	0.001434
GO:0043657	Host cell	DYNLT1, TAP2, TAP1, IFIT1	4	3.54E-05	0.002074
GO:0030669	Clathrin-coated endocytic vesicle membrane	FCGR1B, HLA-DQA2, HLA-DQB2, HLA-DQA1, HLA-DQB1	5	0.003578	0.167455
GO:0042613	MHC class II protein complex	HLA-DQA2, HLA-DQB2, HLA-DQA1, HLA-DQB1	4	0.004496	0.175333
GO:0012507	ER to Golgi transport vesicle membrane	SEC24D, HLA-DQA2, HLA-DQB2, HLA-DQA1, HLA-DQB1	5	0.008384	0.280279
GO:0071556	Integral component of lumenal side of endoplasmic reticulum membrane	HLA-DQA2, HLA-DQB2, HLA-DQA1, HLA-DQB1	4	0.009855	0.288248
GO:0005634	Nucleus	MT1L, MT1X, IFI35, HERC5, SPIC, MT2A, LGALS9, CMTR1, TRIM21, TRIM22, ZNF684, HERC6, DDX17, ZBP1, SP110, RPL22, WDR76, XRN1, HOXB3, MT1G, MT1H, C19ORF66, LAP3, MT1B, DGKH, MT1E, ZNF395, SET, HKR1, SP140, TACSTD2, ZC3HAV1, HESX1, STAP1, SMAD1, PHC2, JUP, PHF11, FANCA, EIF2AK2, PARP14, PTK2, PARP12, ABTB2, ID3, XAF1, APOBEC3A, APOBEC3B, ZNF496, RERE, CLIC5, KDM5D, ADAR, IFIH1, TRIM6, TRIM5, CD38, ZNF366, BATF2, RMI2, DTX3L, SERPINB9, SEPT4, PARP9, ETV7, HSH2D, PLSCR1, AIM2, OAS1, OAS2, IRF7, NCOA7, KPTN, IRF9, CHMP5, SLFN5, SP100, RBM47, SF3B3, DBF4B, USP18, ARVCF, IFI16, HLX, ATOH8, IGF2BP3, DISC1, ZNF467, HNRNPA1, GBP4, STAT1, STAT2, MX2, MX1, NAP1L1, REC8, PML, PSMB9, ZNFX1, TAF8, FAS	101	0.018795	0.48114
GO:0016020	Membrane	LGALS3BP, GRAMD1B, DOCK4, IFITM1, RASL11A, TACSTD2, AGAP1, ADAR, TNFSF13B, OASL, FADS2, CYSLTR1, RNF213, IFI16, LAMP3, KIF5A, ANKFY1, CD38, HNRNPA1, HLA-DQA1, GBP3, APOL2, DDX17, GBP5, PNPT1, C1GALT1, DDHD2, TAP2, TAP1, EIF2AK2, NAP1L1, SERPINB9, PARP9, PARP14, BST2, PLSCR1, GRIN3A, XRN1, OAS2, CDC42EP2, SLC26A8, RPL24, FAS, PAM, HLA-DQB1	45	0.042198	0.759564

**Table 6:** lists the significantly enriched pathways of mild, moderate and severe genes associated with CHE

**KEGG Pathway**	**Name**	**Genes**	**Count**	**P-value**	**FPC**
		**MILD**			
		**-**			
		**MODERATE**			
hsa05164	Influenza A	RSAD2, DDX58, MX1, EIF2AK2, IFIH1, CXCL10, OAS1, OAS2, OAS3, TNFSF10, IRF7, HLA-DQA2, HLA-DQA1, HLA-DQB1	14	9.89E-11	6.33E-09
hsa05168	Herpes simplex infection	IFIH1, OAS1, DDX58, OAS2, OAS3, IRF7, EIF2AK2, IFIT1, HCFC2, HLA-DQA2, HLA-DQA1, HLA-DQB1	12	3.35E-08	1.07E-06
hsa05162	Measles	IFIH1, OAS1, DDX58, OAS2, OAS3, MX1, IRF7, TNFSF10, EIF2AK2	9	3.11E-06	6.64E-05
hsa05160	Hepatitis C	OCLN, OAS1, DDX58, OAS2, OAS3, IRF7, EIF2AK2, IFIT1	8	3.28E-05	5.25E-04
hsa04622	RIG-I-like receptor signaling pathway	IFIH1, CXCL10, DDX58, DHX58, IRF7, ISG15	6	1.15E-04	0.00147
hsa04623	Cytosolic DNA-sensing pathway	ZBP1, CXCL10, AIM2, DDX58, IRF7	5	9.76E-04	0.01037
hsa05310	Asthma	MS4A2, HLA-DQA2, HLA-DQA1, HLA-DQB1	4	0.001134	0.01037
hsa05323	Rheumatoid arthritis	CTSL, TNFRSF11A, HLA-DQA2, HLA-DQA1, HLA-DQB1	5	0.003168	0.02535
hsa05150	*Staphylococcus aureus* infection	C3AR1, HLA-DQA2, HLA-DQA1, HLA-DQB1	4	0.006164	0.04137
hsa04060	Cytokine-cytokine receptor interaction	CX3CR1, CXCL10, CXCL11, TNFSF15, TNFSF10, TNFRSF11A, CCR5	7	0.006464	0.04137
		**SEVERE**			
hsa05164	Influenza A	RSAD2, DDX58, STAT1, STAT2, HSPA6, MX1, EIF2AK2, ADAR, PML, IFIH1, CXCL10, OAS1, OAS2, OAS3, TNFSF10, IRF7, FAS, CCL2, HLA-DQA2, IRF9, HLA-DQA1, HLA-DQB1	22	1.41E-12	2.26E-10
hsa05168	Herpes simplex infection	SP100, DDX58, STAT1, STAT2, TAP2, TAP1, EIF2AK2, IFIT1, PML, IFIH1, OAS1, OAS2, OAS3, IRF7, FAS, CCL2, HLA-DQA2, IRF9, HLA-DQA1, HLA-DQB1	20	2.62E-10	2.11E-08
hsa05162	Measles	DDX58, STAT1, STAT2, HSPA6, MX1, EIF2AK2, ADAR, IFIH1, OAS1, OAS2, OAS3, TNFSF10, IRF7, FAS, IRF9	15	6.01E-08	3.23E-06
hsa05160	Hepatitis C	OCLN, OAS1, DDX58, STAT1, OAS2, STAT2, OAS3, IRF7, EIF2AK2, IFIT1, IRF9	11	9.93E-05	0.004
hsa05150	*Staphylococcus aureus* infection	C1QB, C3AR1, HLA-DQA2, HLA-DQA1, C1QC, C2, HLA-DQB1	7	3.24E-04	0.01044
hsa04623	Cytosolic DNA-sensing pathway	ZBP1, CXCL10, AIM2, DDX58, TREX1, IRF7, ADAR	7	8.17E-04	0.02155
hsa04978	Mineral absorption	MT2A, MT1G, MT1H, MT1X, MT1B, MT1E	6	9.37E-04	0.02155
hsa04622	RIG-I-like receptor signaling pathway	IFIH1, CXCL10, DDX58, DHX58, IRF7, ISG15	6	0.007291	0.14673
hsa04612	Antigen processing and presentation	HSPA6, TAP2, TAP1, HLA-DQA2, HLA-DQA1, HLA-DQB1	6	0.01025	0.18335
hsa05332	Graft-versus-host disease	FAS, HLA-DQA2, HLA-DQA1, HLA-DQB1	4	0.019304	0.31079
